# Cornepalenins A–D, picrotoxane-type sesquiterpenes from the flowers of *Coriaria nepalensis* associated with modulation of GABA_B_ receptor–related signaling

**DOI:** 10.1007/s13659-026-00623-1

**Published:** 2026-08-24

**Authors:** Min Tan, Rong Xie, Dan Liu, Yao Bai, Zhi-Jun Zhang, Rong-Tao Li

**Affiliations:** 1 Faculty of Life Science and Technology, Kunming University of Science and Technology, Kunming, 650500 Yunnan China; 2 Southwest United Graduate School, Kunming, 650092 Yunnan China

**Keywords:** *Coriaria nepalensis*, Picrotoxane-type sesquiterpenes, GABA_B_ receptor signaling, Molecular docking, Network pharmacology

## Abstract

Three new picrotoxane-type sesquiterpenes (PSTs) cornepalenins A–C (**1**–**3**), along with eight known analogues (**4**–**11**), were isolated and identified from the flowers of *Coriaria nepalensis*. In addition, the spectroscopic data and absolute configurations of the known compound cornepalenin D (**4**) are reported for the first time. Biological evaluation results showed that multiple compounds exhibited certain inhibitory effects on GABA_B_ receptor–mediated signaling pathways in both primary cerebellar granule neurons and GABA_B_ receptor–overexpressing HEK 293 T cells. Among them, compound **6** displayed relatively stronger inhibitory activity under stimulation with GABA and baclofen, and was more potent than the positive control CGP54626. The IC_50_ value of compound **6** was 1.41 ± 0.86 μM under GABA stimulation and 1.45 ± 0.55 μM under baclofen stimulation. In addition, mechanistic studies indicated that this compound suppressed the phosphorylation of ERK1/2. Collectively, this study demonstrates that PSTs can modulate GABA_B_ receptor–related signaling pathways and provides experimental evidence for their further investigation in central nervous system–related disorders.

**Graphical Abstract:**

**Figure:**
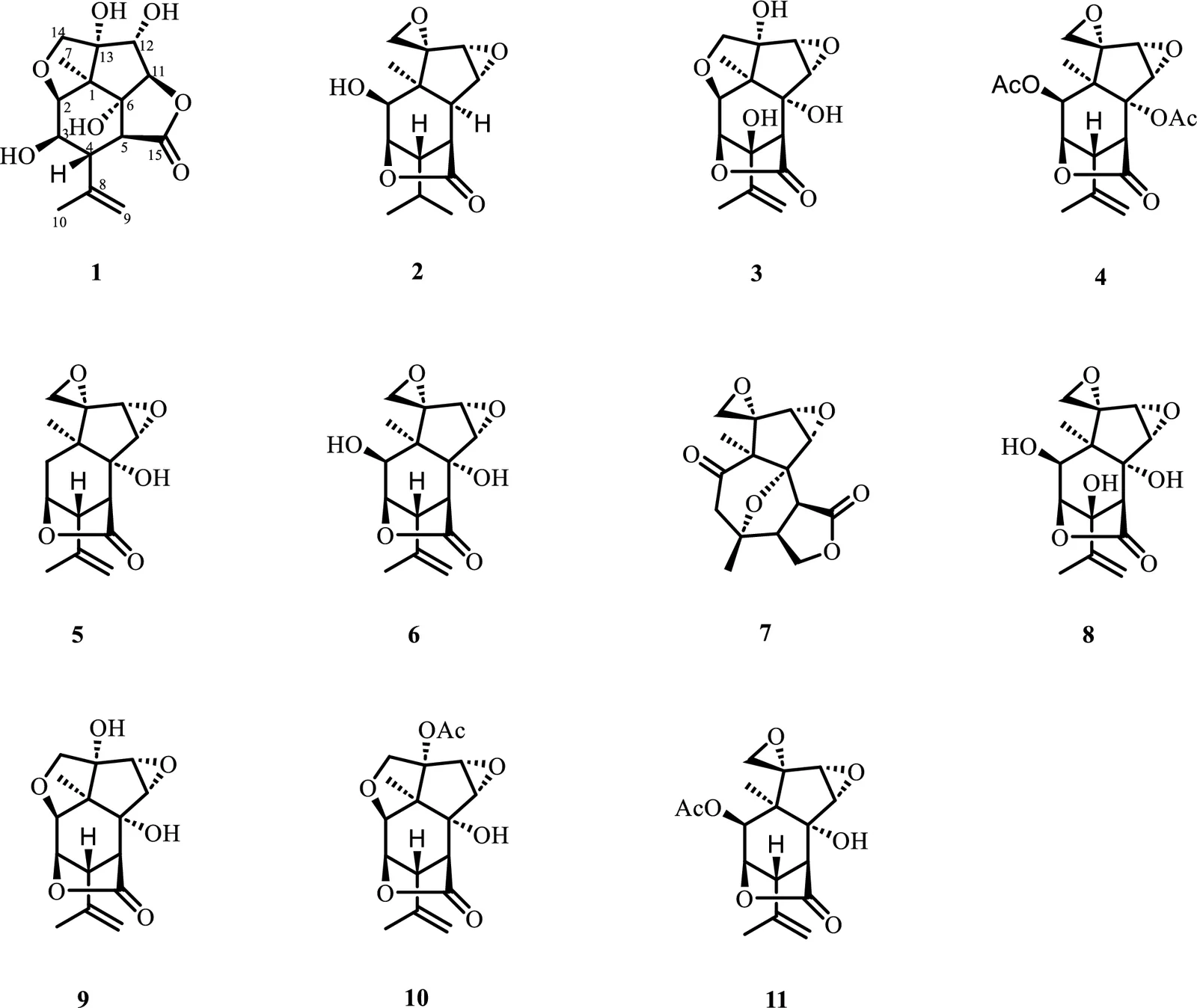

**Supplementary Information:**

The online version contains supplementary material available at 10.1007/s13659-026-00623-1.

## Introduction

Gamma-aminobutyric acid (GABA) represents one of primary inhibitory neurotransmitters found in the central nervous system in mammals [1]. This substance interacts with ionotropic (GABA_A_, GABA_C_) and metabotropic (GABA_B_) receptors [2]. Studies have shown that dysfunction of the GABAergic system plays a significant role in psychiatric disorders, particularly those represented by Alzheimer’s disease (AD) [3]. Research has found that existing GABA_A_ receptor agonists (e.g., muscimol, diazepam) [4–6]. GABA_A_ receptor antagonists (e.g., tetrahydropalmatine, pentylenetetrazol) [7–10] and GABA_B_ receptor agonists (e.g., baclofen) [11] can improve memory and exert neuroprotective effects in AD mice at low doses. However, high doses of these agents may lead to memory impairment and adverse effects such as seizures in AD mice. In a Phase II double-blind, placebo-controlled study involving 110 patients with mild cognitive impairment, once-daily oral administration of 600 mg CGP36742 (a GABA_B_R antagonist) for 8 weeks significantly improved working memory and attention [12]. CGP36742 is currently the first GABA_B_R antagonist to enter clinical trials, and no evidence has yet shown that high doses of GABA_B_R antagonists accelerate the progression of AD. These findings suggest that GABA_B_R antagonists have strong potential as novel therapeutic targets for AD. However, the currently reported agents with GABA_B_ receptor inhibitory function include phaclofen [13], saclofen [14], CGP35348 [15], COR758 [16], and CGP54626 [17], SCH50911 [18], variations in selectivity, potency, and pharmacokinetic properties limit the clinical applications of these antagonists.

Therefore, in the field of drug discovery, the development of novel natural antagonists targeting GABA_B_ receptors holds significant research value. PTX (picrotoxinin: picrotin = 1:1) [19, 20], derived from *Anamirta cocculus*, and tutin, obtained from *Coriaria* (Coriariaceae) species [21], are typical GABA_A_ receptor antagonists. These natural sesquiterpene toxins are closely associated with the central nervous system, however, their potential regulatory mechanisms on GABA_B_ receptors have not yet been systematically investigated. The *Coriaria nepalensis* Wall is widely distributed in southwestern China, and its roots have a long history of use for treating a variety of ailments, such as numbness, toothache, traumatic injuries, and schizophrenia [22]. Picrotoxane-type sesquiterpenes (PSTs) are the characteristic bioactive constituents of family Coriariaceae. Notably, two of these sesquiterpenes, coriamytin and tutin, have been clinically used to treat schizophrenia in China [23], demonstrating the significant potential of these compounds in modulating the central nervous system. Based on this, we attempted a systematic chemical analysis of various parts of *C.nepalensis* (leaves, flowers, and root bark), aiming to isolate more sesquiterpenes and further explore their inhibitory effects on GABA_B_ receptors.

**Fig. 1:**
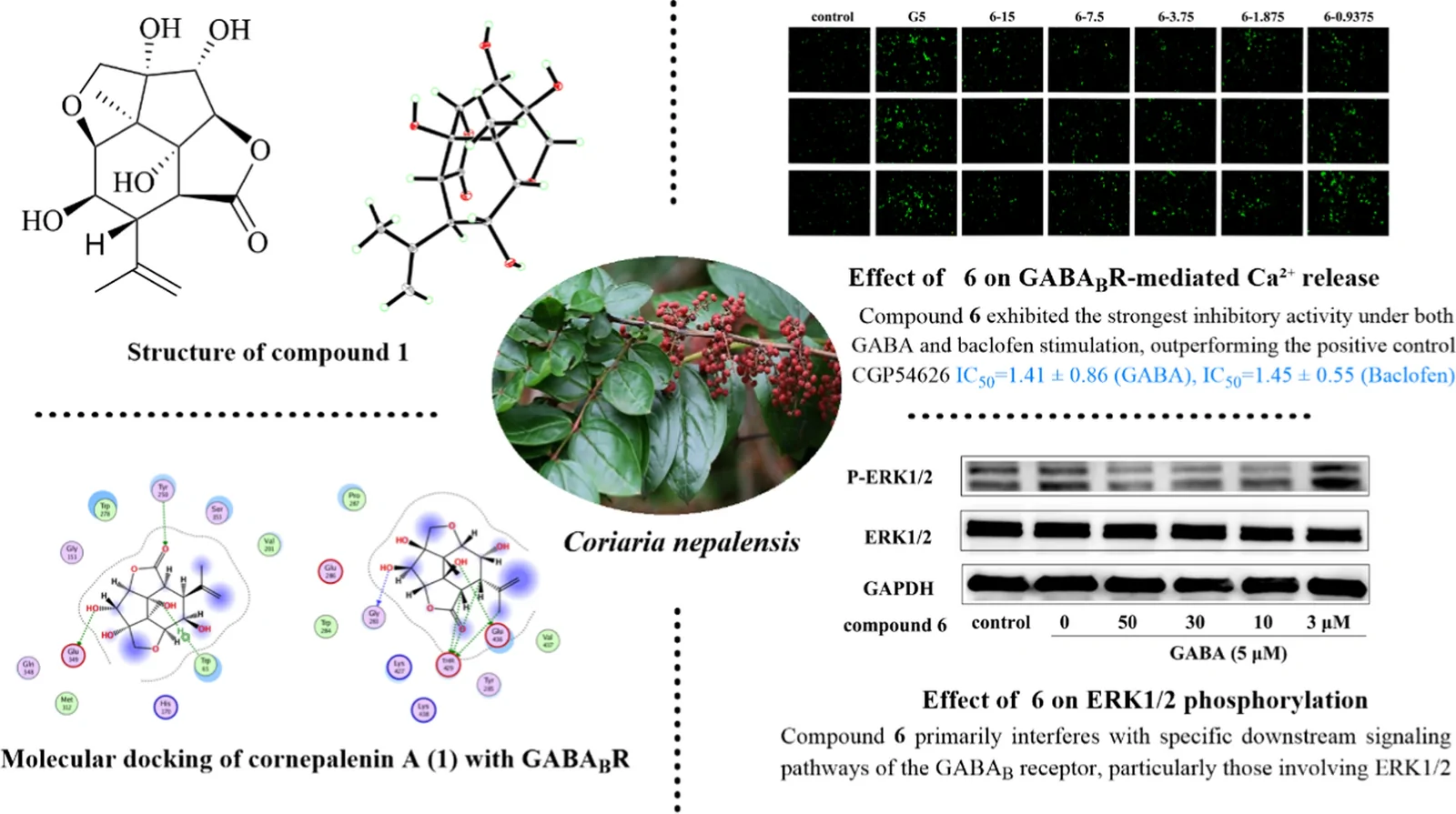
Chemical structures of compounds **1**–**11** from *C. nepalensis*

Initially, a chemical study of the leaves of *C. nepalensis* was conducted, but unfortunately, only one sesquiterpene was isolated [24]. In present study, total eleven PSTs, including three new ones, were isolated (Fig. 1). The molecular docking results suggest that these compounds may exhibit a certain degree of binding affinity toward GABA_A_ receptors, while also showing potential binding capability to GABA_B_ receptors. This finding suggests that PSTs from *C. nepalensis* may not only act as GABA_A_R antagonists, but also potentially function as modulators of GABA_B_R. Based on this, we conducted corresponding experimental verification through GABA_B_R overexpression system in HEK-293 T cells, and the results showed that some of these compounds exhibited inhibitory effects on GABA_B_R signaling pathways.

## Results and discussion

### Structural identification

**Fig. 2:**
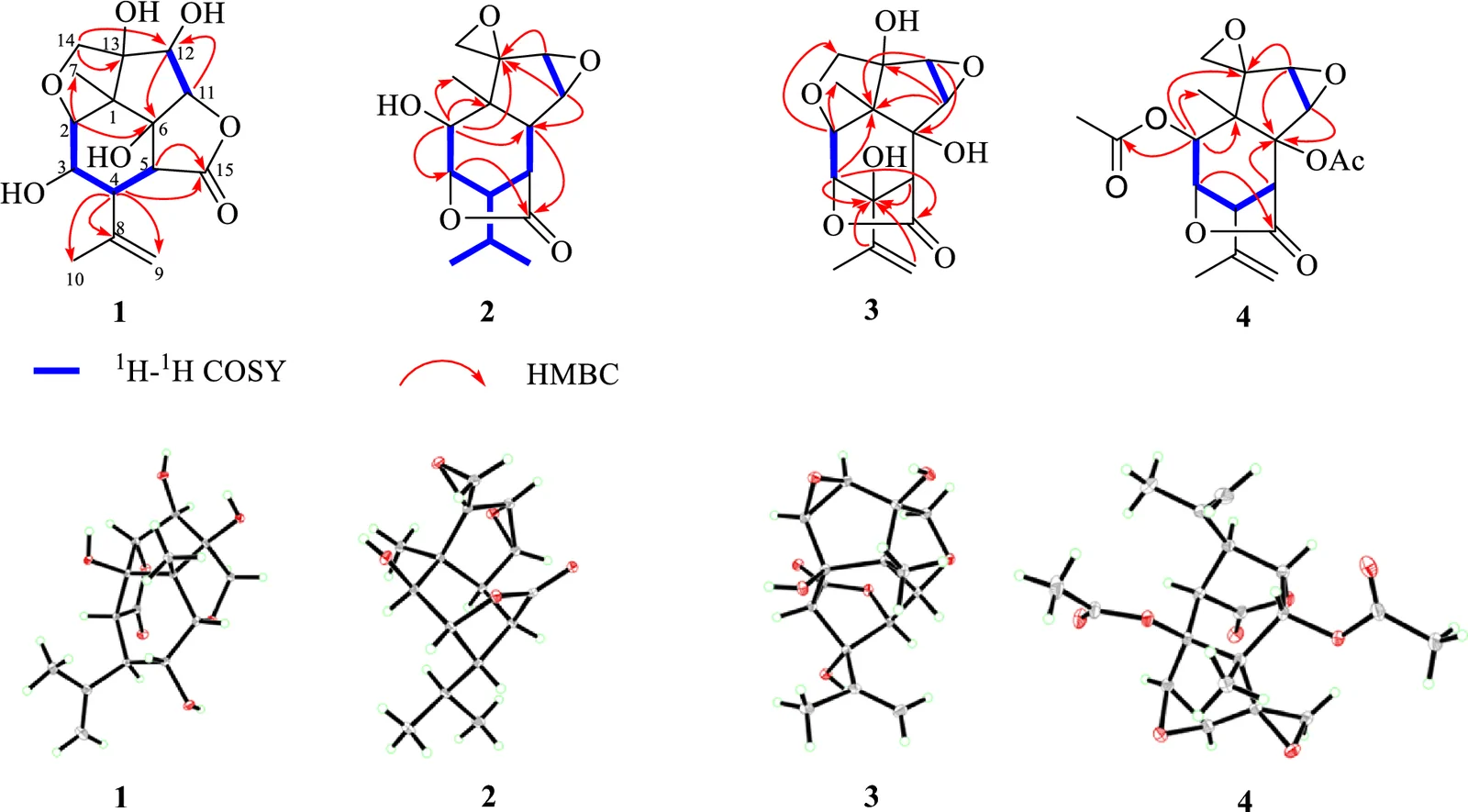
Key HMBC and ^1^H–^1^H COSY correlations, and X-ray ORTEP drawings of compounds **1**–**4**

Cornepalenin A (**1**) was isolated colorless needle crystal. Its positive mode HRESIMS showed the sodium adduct ion peak [M + Na]^+^ at *m*/*z* 335.1111 (calcd. for 335.1101), establishing its molecular formula as C_15_H_20_O_7_. The ^1^H, DEPT, and ^13^C NMR spectra (Table 1) combined with HSQC spectrum exhibited the existence of 15 carbons, consisted of two methyls, an oxygenated methylene, six methines including four oxygen-bearing ones, five quaternary carbons, including two oxygenates, one olefin quaternary carbon and one carbonyl carbon. Comparison of the NMR data above mentioned with corianin [23], indicated their similarity, in the ^1^H NMR spectrum, the H-3 signal of compound **1** shifted upfield, moving from 5.28 (dt, *J* = 4.1 Hz) in corianin to 3.76 (d, *J* = 10.1 Hz), indicating that the lactone ring had hydrolyzed to form a hydroxyl group at C-3. Additionally, the ^13^C NMR spectrum showed that the chemical shift of C-3 moved from 85.4 to 69.4. Meanwhile, the epoxide group formed by C-11 and C-12 in corianin was opened. The C-11 hydrogen signal shifted downfield to 4.32 (d, *J* = 3.6 Hz), compared to 3.84 (d, *J* = 3.0 Hz) in corianin. The C-12 hydrogen signal shifted upfield to 3.83 (d, *J* = 3.1 Hz), and the corresponding C-12 carbon signal shifted from 60.7 to 77.6. These changes indicate that the lactone group moved from C-3 to C-11. Detailed analysis of the HMBC and ^1^H–^1^H COSY spectra (Fig. 2) and combined with ROESY (Figure S1) correlations, the relative configuration of compound **1** was determined. Subsequently, its absolute configuration was established through ECD calculations (Figure S2) and X-ray single-crystal diffraction (0.01(5)) (Fig. 2). Thus, the identification of compound **1** was confirmed.

**Table 1:** ^1^H (600 MHz) and ^13^C (150 MHz) NMR data of compounds (**1**, **3** in CD_3_OD,** 2**, **4** in CDCl_3_)

No	**1**		**2**		**3**		**4**	
	*δ*_H_ (mult, *J*, Hz)	*δ*_C_	*δ*_H_ (mult, *J*, Hz)	*δ*_C_	*δ*_H_ (mult, *J*, Hz)	*δ*_C_	*δ*_H_ (mult, *J*, Hz)	*δ*_C_
1		57.0		40.8		56.2		45.6
2	3.61 m	89.1	3.69 d (2.6)	74.9	4.24 d (3.5)	89.2	5.39 s	74.1
3	3.76 d (10.1)	69.4	4.62 d (5.5)	82.0	4.76 d (3.7)	88.2	4.81 d (4.6)	80.2
4	2.80 dd (10.1, 5.7)	47.4	2.20 dt (10.8, 5.1)	51.2		81.2	3.19 s	49.9
5	2.61 d (5.7)	53.9	2.62 t (3.9)	42.9	2.87 s	57.9	3.90 d (3.9)	46.1
6		83.4	2.41 d (3.3)	42.0		77.2		83.1
7	1.08 s	15.6	1.49 s	26.6	0.94 s	22.7	1.34 s	20.4
8		146.1	1.62 m	25.6		144.1		138.3
9a	4.89 s	114.9	1.00 d (6.0)	21.0	5.01 s	115.1	5.18 s	113.5
9b	4.84 s				5.00 s		5.07 s	
10	1.76 s	19.7	0.98 d (6.0)	19.8	1.92 s	20.1	1.76 s	22.4
11	4.32 d (3.6)	93.3	3.69 d (2.6)	56.3	3.74 d (2.4)	64.4	3.16 d (3.0)	60.1
12	3.83 d (3.1)	77.6	3.15 d (3.0)	59.6	3.43 d (2.4)	61.3	4.61 d (3.0)	56.9
13		89.7		66.9		91.4		64.3
14a	3.80 d (9.9)	76.0	4.05 d (4.9)	52.4	3.96 d (10.4)	78.3	3.76 d (4.9)	52.6
14b	3.41 d (9.9)		2.83 d (4.9)		3.77 d (10.4)		2.88 d (4.9)	
15		178.5		177.1		176.7		173.4
2′								170.7
2′′							2.13 s	21.0
6′								170.4
6′′							2.09 s	21.7

Cornepalenin B (**2**) was obtained in the form of a white amorphous powder and its molecular formula was determined to be C_15_H_20_O_5_ based on the positive-ion HRESIMS at *m*/*z* 303.1209 [M + Na]^+^ (calcd. for 303.1203). The ^1^H NMR spectrum (Table 1) of compound **2** displayed signals for three methyl singlets, one oxygenated methylene, four oxygenated methine proton resonance. The ^13^C NMR and DEPT spectra (Table 1) depicted 15 carbon resonances, consisting of three methyl, one methylene (oxygenated), eight methines (four oxygenated), and three quaternary carbons (one ester carbonyl, one oxygenated). Based on these NMR data, it is speculated that **2** is a picrotoxane-type sesquiterpene, and these data indicated that compound **2** was chemically similar with the known analogue, corialactone A [25]. The primary distinction between the two compounds was observed in the C-2 chemical shift [*δ*_C_ 31.4 in corialactone A; *δ*_C_ 74.9 in **2**], implying that the methylene at C-2 in corialactone A may be replaced by an oxygen-containing methine group. This hypothesis was validated through HMBC and ^1^H–^1^H COSY correlations (Fig. 2). Additionally, the relative configuration of compound **2** was determined using ROESY experiments (Figure S1). Furthermore, the absolute configuration of compound **2** was determined through ECD calculations (Figure S2) and X-ray single-crystal diffraction (0.10(2)) (Fig. 2) analysis. Hence, compound **2** was identified.

Cornepalenin C (**3**) was acquired as colorless needle crystal, with a molecular formula determined to be C_15_H_18_O_7_ from the quasi-molecular ion peak at *m*/*z* 333.0941 [M + Na]^+^ (calc. for 333.0945) in the HRESIMS. The ^1^H and ^13^C NMR (Table 1) spectral data of **3** closely resembled those of corianin [23]. Comparison of the NMR data mentioned above with those of corianin revealed striking similarities, except for the presence of a hydroxyl group at C-4 in compound **3**, which resulted in a downfield shift of C-4 (δ_C 49.6 → 81.2). The presence of a hydroxyl group at C-4 was unequivocally confirmed through HMBC correlations (Fig. 2). Additionally, ROESY correlations (Figure S1) established that all chiral centers in compound **3** share the same relative configuration as those in compound **2**. The absolute configuration of compound **3** was determined through ECD calculations (Figure S2) and single-crystal analysis (0.04(8)) (Fig. 2). Thus, the structure of compound **3** was identified.

Cornepalenin D (**4**) was acquired as a white amorphous powder and established to possess a molecular composition of C_19_H_22_O_8_ according to the HRESIMS (*m*/*z* 401.1206 [M + Na]^+^, calc. for 401.1207). The signals depicted in the ^1^H NMR (Table 1) of **4** included four methyl groups, an olefinic methylene, and one oxygenated methylene, four oxygenated methine proton resonances. The ^13^C NMR (Table 1) and DEPT spectra displayed 15 carbon signals with four methyls, two methylenes containing an olefinic one, six methylenes, with four oxygen signals. Additionally, there were three oxygenated quaternary carbons, which included a carbonyl carbon at *δ*_C_ 173.4 (C-15). The carbon signals observed in the ^13^C NMR spectroscopic spectrum of **4** bore a striking resemblance to those of a known compound, tutin [23]. The primary distinction between them was the shift of C-2 (*δ*_C_ 72.2–74.1) and C-6 (*δ*_C_ 77.9–83.1) down-field in **4**, resulting from the replacement of the hydroxyl groups of C-2 and C-6 with two acetyl groups in **4**. This change was further supported by the molecular formula of **4**. The structure was confirmed based on its HRESIMS data and HMBC correlations (Fig. 2). Additionally, analysis of the ROESY spectrum (Figure S1) determined that compound **4** shares the same relative configuration as compound **2**. Subsequently, the absolute configuration of compound **4** was determined through ECD calculations (Figure S2) and single-crystal analysis (−0.04(4)) (Fig. 2). Therefore, the structure of compound **4** was ultimately identified.

The known compounds were identified included coriamyrtin (**5**) [23], tutin (**6**) [23], corianlactone (**7**) [26], hyenanchin (**8**) [27], corianin (**9**) [23], acetylcorianin (**10**) [23], acetyltutin (**11**) [28], using NMR data by comparison with literature values.

### Molecular docking analys

**Fig. 3:**
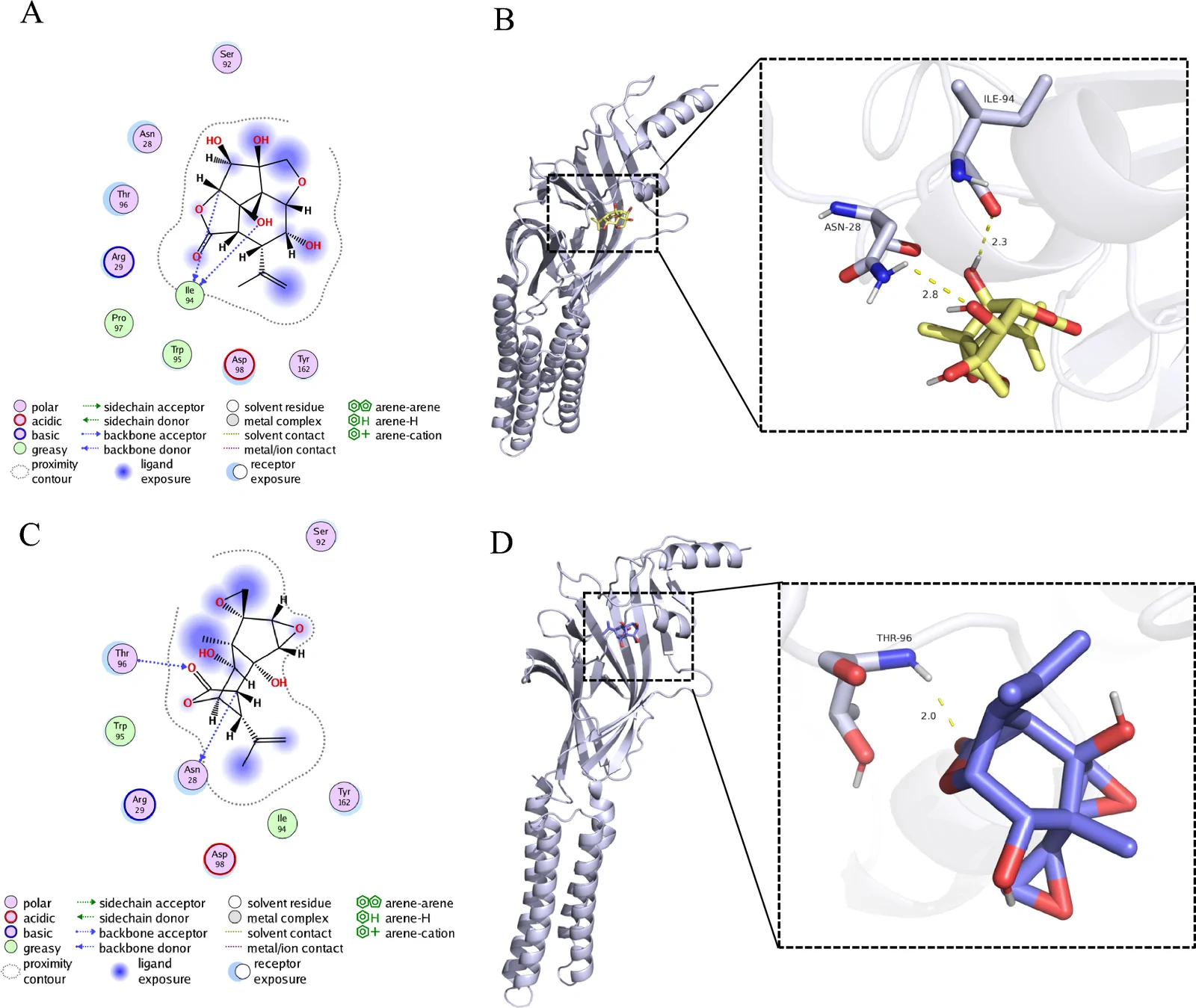
2D and 3D representations of compounds **1** and **6** showing their interactions with the GABA_A_ receptor. **A**, **C** 2D interaction diagram of **1, 6** with GABA_A_R1; **B**, **D** Predicted binding conformation of **1**, **6** within the 3D structure of GABA_A_R1

**Fig. 4:**
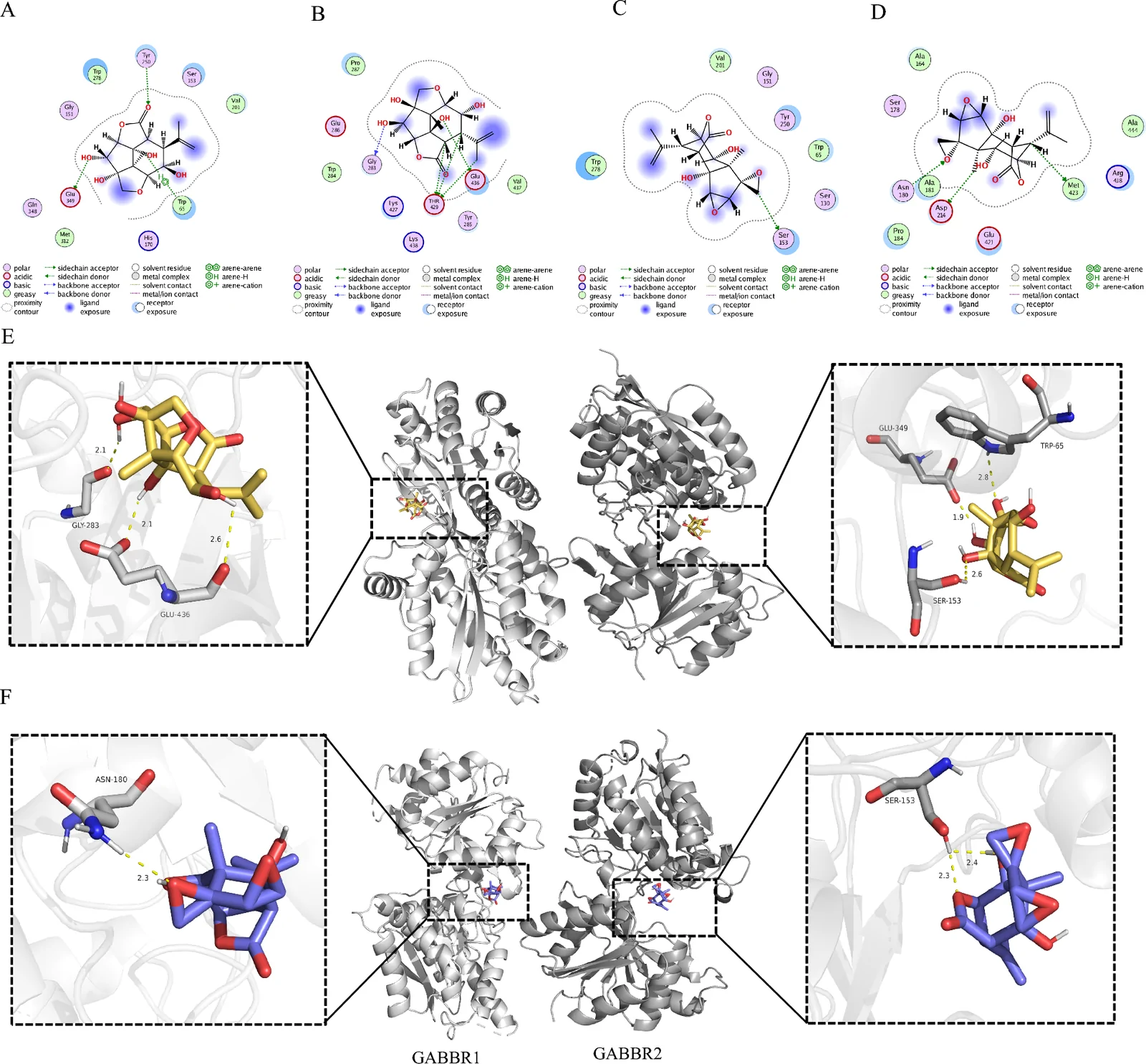
2D and 3D representations of compounds **1** and **6** showing their interactions with the GABA_B_ receptor. **A**, **B** 2D interaction diagrams of compound **1** with GABBR1and GABBR2, respectively. **C**, **D** 2D interaction diagrams of compound **6** with GABBR1and GABBR2, respectively. **E**, **F** Predicted binding conformations of compounds **1** and **6** within the 3D structure of the GABBR1and GABBR2 complex

Molecular docking is a widely used computational approach for investigating ligand–protein interactions. In this study, we focused on the GABA_A_ and GABA_B_ receptor proteins to evaluate the binding affinities and interaction patterns of this class of compounds. The docking simulations were performed using the crystal structures of GABA_A_R1 (PDB ID: 8VQY), GABBR1 (PDB ID: 4MQF), and GABBR2 (PDB ID: 4MR7), and the resulting conformations are presented in Figs. 3 and 4. Compound **1** formed two hydrogen bonds with residues ILE94 and ASN28 in the GABAAR1 receptor, with bond lengths of 2.3 Å and 2.8 Å, respectively, and a binding energy of −5.8 kcal/mol. Compound **6** interacted with residue THR96 via a single hydrogen bond (2.0 Å), showing a slightly higher binding affinity with an energy of −5.9 kcal/mol. In the case of the GABA_B_ receptors, compound **1** formed three hydrogen bonds with residues TRP65, GLY349, and SER153 of GABBR1, at distances of 2.8 Å, 2.6 Å, and 1.9 Å, respectively, with a binding energy of −6.6 kcal/mol.

Additionally, it formed three hydrogen bonds with residues GLY283 and GLU436 of GABBR2 (bond lengths: 2.1 Å, 2.1 Å, and 2.6 Å), yielding a binding energy of −6.8 kcal/mol. Compound **6** formed two hydrogen bonds with SER153 in GABBR1 (2.3 Å and 2.4 Å) and one hydrogen bond with ASN180 in GABBR2 (2.3 Å), with binding energies of −6.5 and −6.9 kcal/mol, respectively. Overall, the results suggest that both compounds **1** and **6** effectively occupy the active sites of GABA_A_R and GABA_B_R receptors, exhibiting favorable binding energies and stable interactions. These findings indicate that compounds **1** and **6** have potential as GABA receptor antagonists, providing strong support for their further development in the context of drug discovery for neurological disorders.

### Bioactivity evaluation

**Fig. 5:**
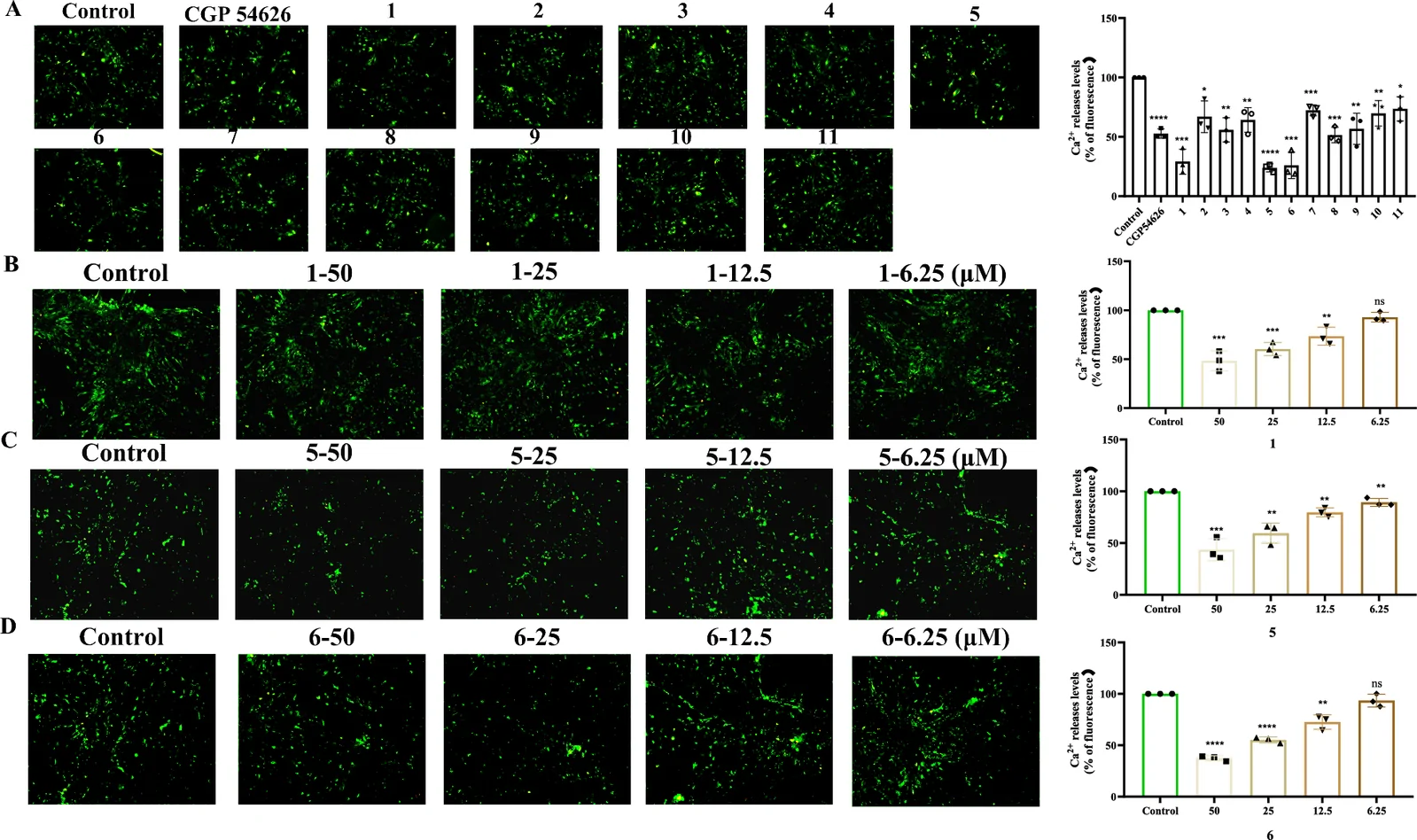
Effects of compounds **1**–**11** on Ca^2^⁺ release in CGNs. **A** Representative fluorescence images and corresponding bar graph quantification showing intracellular Ca^2^⁺ levels in CGNs treated with CGP54626 (50 μM) and compounds **1**–**11** (50 μM). Fluorescence intensity is expressed as a percentage of the untreated control group (100%). **B**–**D** Representative fluorescence images and bar graphs showing dose-dependent effects of compounds **1** (**B**), **5** (**C**), and **6** (**D**) on Ca^2^⁺ release at concentrations of 50, 25, 12.5, and 6.25 μM. Scale bars:100 μm. Quantitative data are presented as percentages relative to the control group. All values are expressed as mean ± SEM (n = 3). Statistical significance is indicated as *p < 0.05, ** *p* < 0.01, ***p < 0.001, and ****p < 0.0001

Firstly, all isolates were evaluated for their effects on GABA_B_ receptor signaling in endogenous primary cerebellar cells of C57BL/6 neonatal mice [29]. The experimental results revealed that at a concentration of 50 μM, compounds **1**–**11** and the positive control CGP54626 significantly reduced intracellular calcium flux signals (Fig. 5A). Given that specific inhibition of GABA receptors may affect intracellular Ca^2^⁺ levels, these results suggest that the tested compounds may attenuate GABA-induced downstream Ca^2^⁺ signaling responses and are associated with inhibitory effects on GABA_B_ receptor activation. Therefore, compounds **1**, **5**, and **6**, which showed more pronounced effects in the initial screening, were selected for further concentration-gradient experiments. The experimental results (Fig. 5B–D) demonstrated that as the concentration of the compounds increased, the activation of the GABA_B_ receptor gradually decreased, with intracellular calcium flux signals showing a distinct concentration-dependent reduction. This suggests that compounds **1**, **5**, and **6** exhibit a dose-dependent inhibitory effect on the GABA_B_ receptor.

**Fig. 6:**
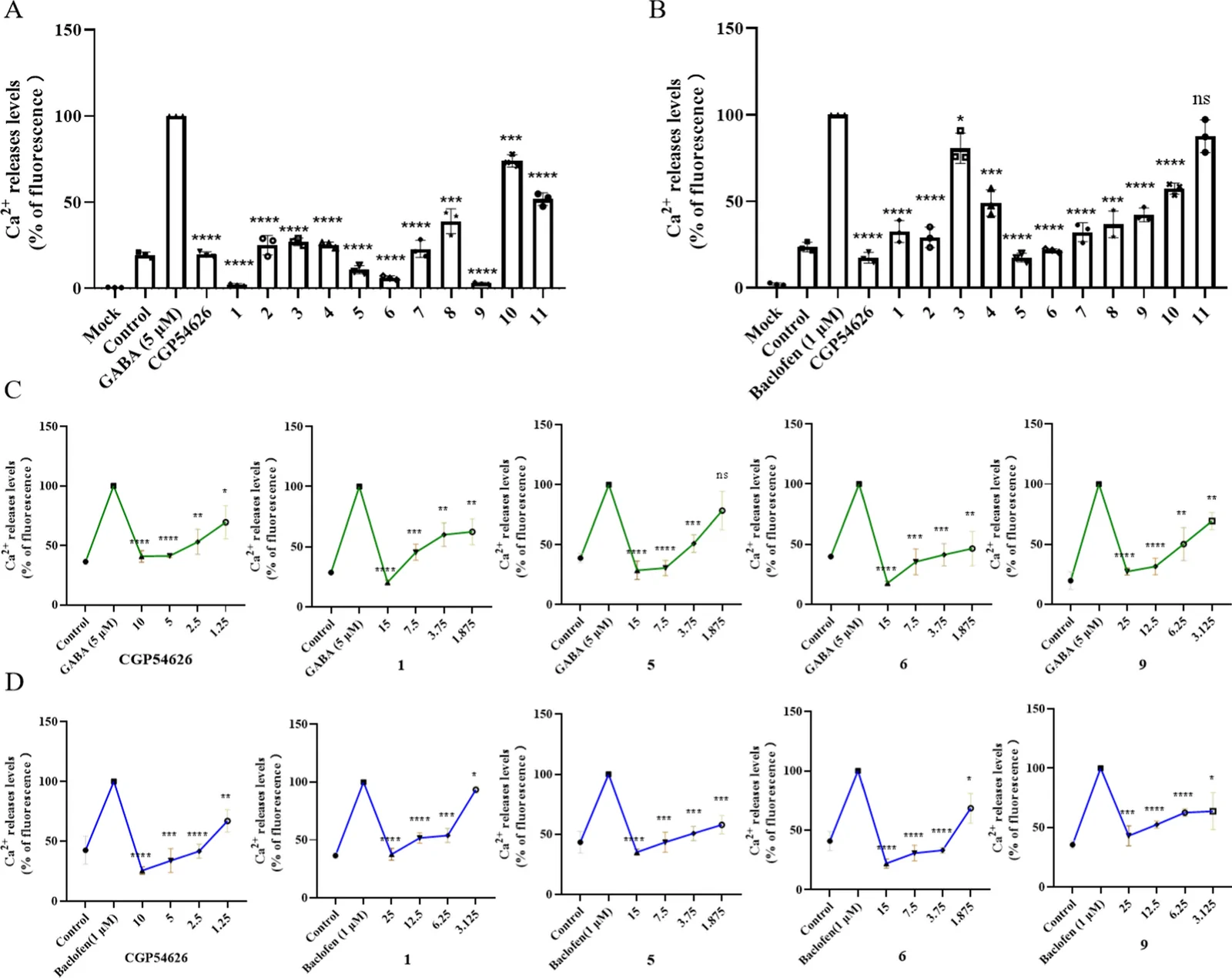
Effects of compounds **1**–**11** on GABA_B_R-mediated Ca^2^⁺ release in 293 T cells under stimulation with the endogenous agonist (GABA, 5 μM) and the selective agonist (baclofen, 1 μM), and dose–response analysis. **A**, **B** Ca^2^⁺ release measured by fluorescence intensity after treatment with CGP54626 (50 μM) and compounds **1**–**11** (50 µM). **C** Dose–response curve of CGP54626 and compounds **1**, **5**, **6**, and **9** on Ca^2^⁺ release under GABA stimulations; **D** Dose–response curve of CGP54626 and compounds **1**, **5**, **6**, and **9** on Ca^2^⁺ release under baclofen stimulations, with fluorescence intensity expressed as a percentage of the agonist group. Statistical significance is indicated as *p < 0.05, **p < 0.01, ***p < 0.001, ****p < 0.0001

Subsequently, GABA_B_ receptor overexpression system in HEK-293 T cells has been successfully established (Figure S3) [29]. Under GABA and Baclofen stimulation, the effects of compounds **1**–**11** on intracellular Ca^2^⁺ release levels were measured (Fig. 6A, B). The experimental results showed that compounds **1**–**11** significantly inhibited intracellular Ca^2^⁺ release levels in response to both agonists. Based on the initial screening results, compounds **1**, **5**, **6**, and **9** were selected for further concentration gradient testing. Under GABA and Baclofen stimulation, the effects of these compounds at different concentrations on GABA_B_ receptor activation were analyzed (Fig. 6C, D). Results demonstrated that as the concentration of the compounds increased, GABA_B_ receptor activation gradually decreased, with intracellular Ca^2^⁺ signals exhibiting a significant concentration-dependent reduction.

This indicates that compounds **1**, **5**, **6**, and **9** exhibit dose-dependent inhibitory effects on GABA_B_ receptor activation. Additionally, concentration-dependent inhibitory curves were statistically analyzed for compounds **1**, **5**, **6**, and **9**, as well as the positive control CGP54626, to calculate their IC_50_ values (Table 2) (IC_50_ refers to the concentration of a drug required to achieve 50% inhibitory effect, reflecting the compound's inhibitory potency against the agonists). The results showed that compound **6** exhibited the strongest inhibitory activity under both GABA and Baclofen stimulation, outperforming the positive control CGP54626. The IC_50_ of compound **6** was 1.41 ± 0.86 μM under GABA stimulation and 1.45 ± 0.55 μM under baclofen stimulation.

**Table 2:** IC₅₀ values (mean ± SD) of compounds **1**, **5**, **6**, and **9** and the positive control CGP54626 under stimulation with GABA (5 μM) and Baclofen (5 μM)

Compounds	CC_50_ (*μ*M)	IC_50_ (*μ*M)	
		GABA (5* μM*)	Baclofen (5* μM*)
**1**	> 50	7.72 ± 2.38	5.61 ± 1.03
**5**	> 50	2.74 ± 0.91	1.99 ± 0.30
**6**	> 50	1.41 ± 0.86	1.45 ± 0.55
**9**	> 50	3.32 ± 0.30	6.43 ± 0.81
CGP54626	> 50	2.04 ± 0.16	1.50 ± 0.53

**Fig. 7:**
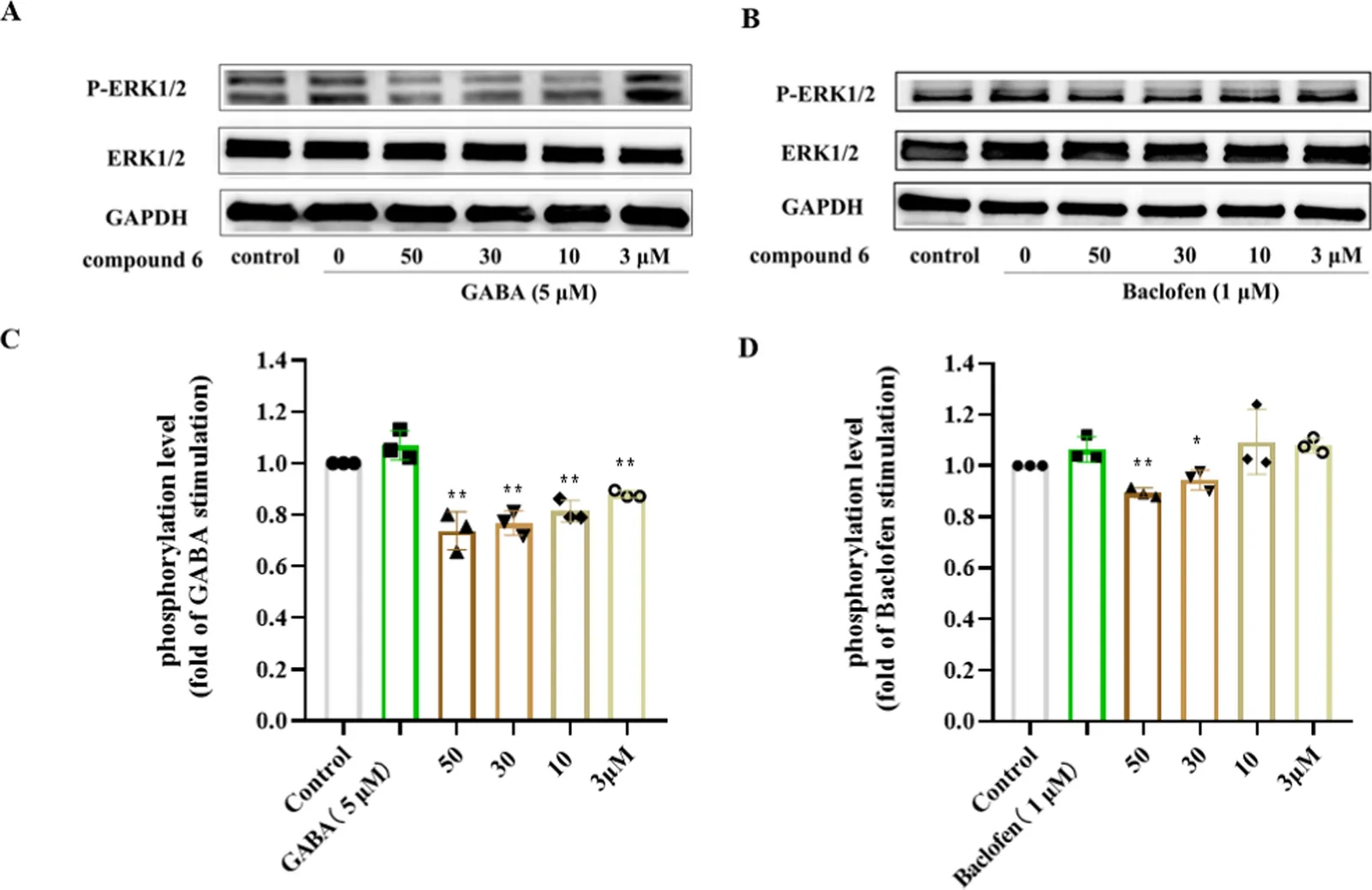
Effect of compound **6** on ERK1/2 phosphorylation induced by GABA or baclofen in transfected HEK 293 T cells. **A**, **B** Western blot analysis of p-ERK1/2 and total ERK1/2 in HEK 293 T cells treated with compound **6** (50, 30, 10, 3 µM) under GABA (5 µM) or baclofen (1 µM) stimulation, with GAPDH as a loading control. **C**, **D** Quantification of p-ERK1/2 levels expressed as a percentage of the corresponding agonist-treated group. Statistical significance is indicated by asterisks (*p < 0.05, **p < 0.01)

Activation of the GABA_B_ receptor triggers a network of signaling pathways, including the activation of key downstream molecules ERK1/2 and AKT, leading to elevated phosphorylation levels. These phosphorylated signaling molecules play essential roles in physiological processes such as neuronal protection, synaptic plasticity, and neurotransmitter transmission by mediating critical pathways. To further investigate the downstream effects of compound **6**, the most active compound identified, we evaluated its impact on the phosphorylation of ERK1/2 and AKT1 in an exogenous system. The experimental results (Fig. 7) showed that compound **6** significantly inhibited the phosphorylation levels of ERK1/2 induced by the agonists Baclofen and GABA, indicating its ability to regulate GABA_B_ receptor-mediated ERK1/2 activation. However, under the same conditions, compound **6** did not exhibit a significant inhibitory effect on AKT1 phosphorylation levels (Figure S50). Compound **6** selectively inhibited GABA_B_ receptor-mediated phosphorylation of ERK1/2, while showing minimal impact on AKT1 phosphorylation. This suggests that compound **6** primarily interferes with specific downstream signaling pathways of the GABA_B_ receptor, particularly those involving ERK1/2.

### Network pharmacology

**Fig. 8:**
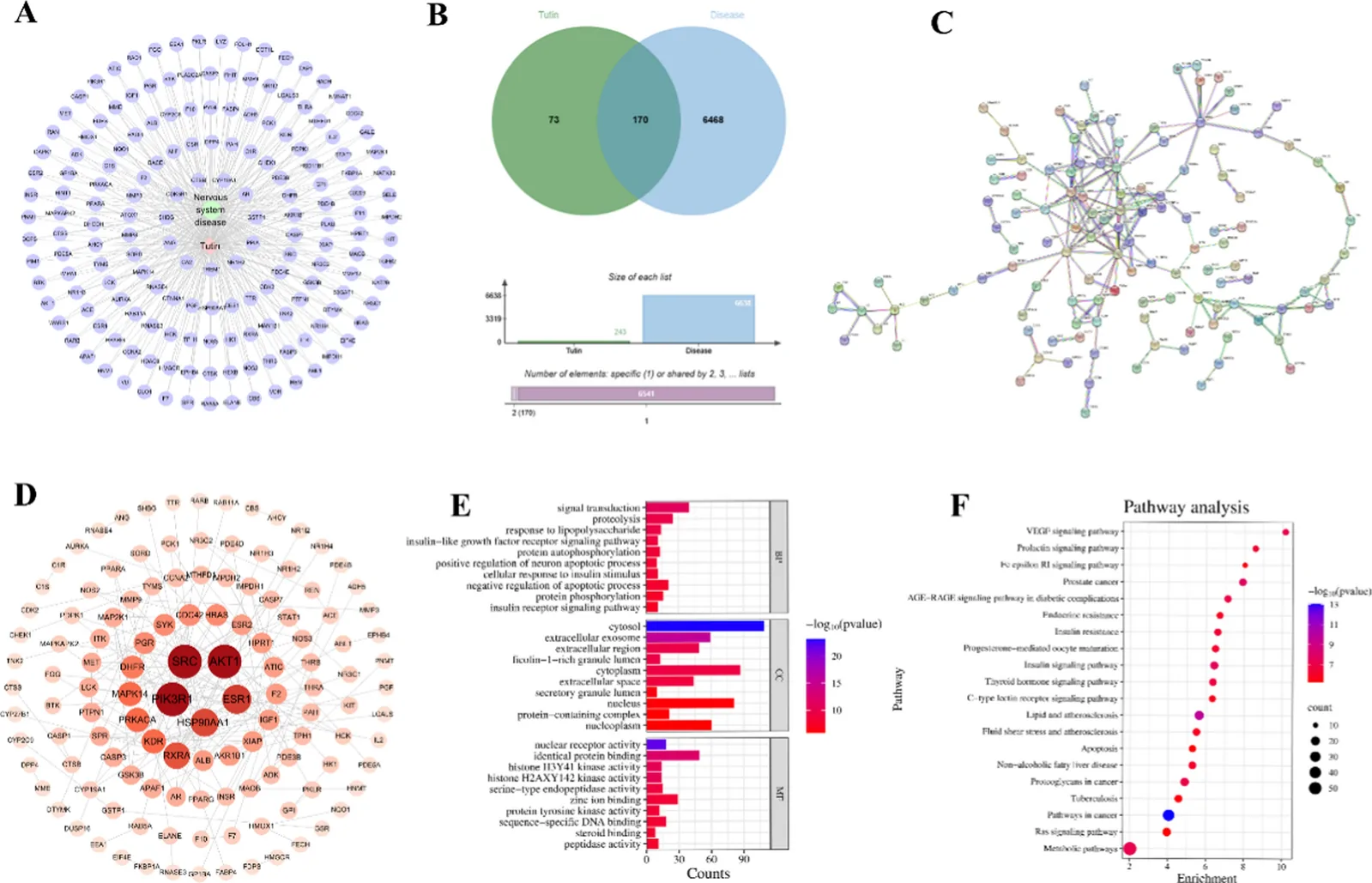
Network pharmacology predicts the targets of compound **6** for the treatment of nervous system diseases. **A** Compound-target-disease association map. **B** Venn diagram of drug targets and nervous system disease targets. **C** PPI (protein–protein interaction) network diagram. **D** PPI (protein–protein interaction) network diagram. **E** GO enrichment analysis of active targets for drug treatment of nervous system diseases. **F** KEGG metabolic pathway enrichment analysis diagram

Targets of compound **6** in neurological diseases were predicted and verified through network pharmacology. The network diagram (Fig. 8A) shows that compound **6** is associated with multiple targets (e.g. AKT1, MAPK14, BACE1, and GSK3β), suggesting it may regulate neurological diseases via multi-target mechanisms. Core targets like AKT1 and GSK3β play key roles in disease regulation. Venn diagram analysis (Fig. 8B) demonstrates that 170 of the 243 targets of compound 6 intersect with disease-related targets. PPI network analysis (Fig. 8C, D) highlights key proteins such as AKT1, MAPK14, GSK3β, and STAT3 in treating neurological diseases. GO enrichment analysis (Fig. 8E) shows that compound **6** may affect biological processes like signal transduction and neuronal apoptosis, cellular components like cytosol and exosomes, and molecular functions like nuclear receptor activity and protein tyrosine kinase activity. KEGG pathway analysis (Fig. 8F) suggests that targets of compound **6** may influence neuroprotection, neuroinflammation, and metabolism via pathways like VEGF, insulin signaling, and apoptosis.

## Discussion and conclusion

In summary, the chemical constituents of the ethanol extract from the flowers of *C. nepalensis* were studied, which resulting in the isolation and identification of eleven PSTs, including three previously unreported. Molecular docking analyses were performed to explore the potential target characteristics of these compounds, and the results suggested that they may have potential interactions with both GABA_A_ and GABA_B_ receptor systems. Bioactivity assays demonstrated that compounds **1**, **5**, **6**, and **9** inhibited GABA_B_ receptor activation to varying degrees, supporting their potential involvement in GABA_B_-related signaling processes. Mechanistic studies further showed that compound **6** markedly attenuated GABA_B_ receptor–mediated ERK1/2 phosphorylation, suggesting that it may play a regulatory role in downstream signaling pathways associated with GABA_B_ receptor activation. Notably, network pharmacology analysis provided important evidence for clarifying the potential mechanistic link between computational predictions and experimental validation. The results showed that the predicted targets of compound **6** were significantly enriched in multiple MAPK/ERK-related signaling pathways, including the PI3K–AKT pathway, cAMP signaling, and Ca^2^⁺-dependent pathways. These pathways exhibit extensive crosstalk and convergence within cellular signaling networks and collectively regulate the activation status of ERK1/2 as a key downstream effector, which is consistent with and supportive of the experimentally observed decrease in ERK1/2 phosphorylation. Therefore, the network pharmacology predictions and the ERK1/2-related experimental findings demonstrate strong mechanistic consistency and mutual support, thereby enhancing the overall rationality and credibility of the conclusions of this study.

In addition, PSTs, as characteristic constituents of plants in the Coriariaceae family, have long been recognized for their modulatory effects on the central nervous system in traditional medicine. For instance, tutin and coriamytin have been historically used to treat mental disorders [22]. This study not only verifies their influence on the GABA_B_ signaling pathway from a modern pharmacological perspective but also provides scientific support for the contemporary development of these traditionally used medicinal plants. Furthermore, molecular docking and network pharmacology analyses revealed that compound **6** forms stable hydrophobic and hydrogen-bond interactions with the active site of the GABA_B_ receptor, further indicating its potential to act as a putative receptor antagonist. These findings lay a theoretical foundation for future semi-synthetic modifications based on natural scaffolds to optimize the pharmacodynamic and pharmacokinetic properties of GABA_B_ receptor antagonists.

In conclusion, this study not only expands the pharmacological profile of PST-type compounds but also, for the first time, establishes their functional relevance to the GABA_B_ receptor, highlighting the great potential of this structural class as lead compounds for the treatment of neuropsychiatric disorders.

## Experimental

### General experimental procedures

ESIMS and HRESIMS were performed by API-Qstar-TOF instrument (Applied Bio-system Corporation, Canada) or LC-IT-TOF instrument (AB-MDS Sciex, Concord, ON, Canada). NMR spectra were obtained using a Bruker AV 600 MHz spectrometer with tetramethylsilane (TMS) as an internal standard (Bruker BioSpin Group, Germany). Optical rotations were determined by a Jasco DIP- 370 digital polarimeter (JASCO Corporation, Tokyo, Japan). UV spectra were measured on a Shimadzu UV-2401A spectrophotometer (Shimadzu Corporation, Tokyo, Japan). Single-crystal X-ray diffraction data were collected on a D8 VENTURE single-crystal X-ray diffractometer (Bruker, Germany). ECD spectra of samples disproved in MeOH were recorded by V100 Chirascan instrument (cells: 1 mm, time-per-point: 1 s/nm (25 μs × 40,000, 195–400 nm), Applied Photophysics Limited, England). Semipreparative HPLC was conducted on an Agilent 1200 liquid chroma-tography (Zorbax SB-C18 column, 5 μm, 9.4 mm × 250 mm, Agilent, USA). Column chromatography (CC) was carried out on silica gel (200–300 mesh, Qingdao Marine Chemical Co. Ltd., P. R. China), MCI gel (CHP 20P, 75–150 μm, Mitsubishi Chemical Corporation, Tokyo, Japan), ODS-C18 (75 μm, YMC Co., Ltd., Japan) and Sephadex LH-20 (Amersham Biosciences AB, Uppsala, Sweden). Fractions were monitored by TLC plates (silica gel GF254, Qingdao Marine Chemical Co. Ltd., P. R. China). Visualization of spots was under UV light (254 nm) or by spraying with 5% H_2_SO_4_–EtOH followed by heating. All other experimental procedures were performed as previously reported [27].

### Plant material

The flowers of *C. nepalensis* were picked from Lijiang City, Yunnan Province, China (N27°28′, E100°85′), and authenticated by Dr. Xuan-Qin Chen. A voucher specimen (number: KUST20220715) was preserved at the Faculty of Life Science and Technology, Kunming University of Science and Technology.

### Extraction and isolation

The dried flowers of *C. nepalensis* (11.0 kg) were extracted with 95% aq. EtOH (each 25 L, 24 h) three times to yield the crude extract (1.5 kg). Next, the EtOH fraction was suspended in water and combined with ethyl acetate (EtOAc) (3 × 25L) to yield an EtOAc extract (500.0 g), This extract was then subjected to silica gel column chromatography using a stepwise gradient of petroleum ether (PE)/acetone (50:0 to 1:1, v/v), resulting in the separation of eight fractions (Fr. A–H). Separation of Fr. D (36.9 g) by reversed-phase column chromatography, eluting with a gradient of MeOH/H_2_O (60:40 to 100:0, v/v) obtained eight subfractions (Fr. D1–D8). Fr. D2 (131.9 mg) was recrystallized in acetone to give compound **5** (3.5 mg). Fr. E (130.0 g) was fractionated using ODS CC with a stepwise gradient system of MeOH/H_2_O (30:70, 50:50, 70:40, 70%, 90%, 100%, v/v) leading to the generation of seven subfractions (Fr. E1–E7). Compound **6** (2.6 g) was obtained from Fr. E1 (5.1 g) through recrystallization in chloroform. The remaining portion of Fr. E1 (2.5 g) underwent silica gel CC (CH_2_Cl_2_/MeOH, 40:1 to 5:1, v/v) to produce eight subfractions (Fr. E1.1-E1.8). Urther purification of Fr. E1.2 (25.0 mg) over silica gel with CH_2_Cl_2_/MeOH (30:1, v/v) resulted in the isolation of compound **7** (7.1 mg). Fr. E1.3 (47.0 mg) was subjected to silica gel CC (PE/acetone, 6:1, v/v) to yield compound **8** (10.0 mg). Fr. E1.6 (390.0 mg) was fractionated using CC over silica gel (CHCl_3_–MeOH, gradient system: 10:1–4:1) to give four fractions Fr E1.6.1–Fr E1.6.4. Fr. E1.6.2 (78.0 mg) was further purified on a Sephadex LH-20 column (MeOH) to yield compounds **4** (10.0 mg) and **10** (12.0 mg). Fr. E2 (3.6 g) was recrystallized in chloroform to obtain compound **9** (750.0 mg). Chromatography of Fr. E2.2 (2.6 g) on silica gel CC (CH_2_Cl_2_/MeOH, 40:1 to 10:1, v/v) to generated six subfractions, E2.2.3 (78.0 mg) was subjected to silica gel CC (PE/acetone, 30:1, v/v), and Sephadex LH-20 CC (MeOH) to give compound **3** (4.2 mg). Compound **2** (8.0 mg, t_R_ 16.8 min) was obtained from Fr. E3 (1.3 g) through silica gel CC (CHCl_3_ /MeOH, 50:1 to 10:1, v/v), then followed by silica gel CC (PE/EtOAc, 1:1, v/v) and further separated by semi-preparative HPLC (30% MeOH/H_2_O). Fr. F (52.8 g) was applied to silica gel using a CH_2_Cl_2_/MeOH, gradient (70:1 to 3:1 v/v) to yield nine subfractions (Fr. F1–F9). Fr. F5 (3.0 g) was separated into four subfractions (Fr. F5.1–F5.4) by a silica gel column using a gradient of CHCl_3_/MeOH (8:1 to 2:1, v/v). Fr. F5.2 (1.2 g) was further purified by repeated silica gel column chromatography (CHCl_3_/MeOH, gradient, 10:1 to 3:1, v/v) and recrystallized in acetone to obtain compound **1** (7.0 mg). Compound **11** (9.0 mg) was isolated from Fr. F7 (1.3 g) through a series of silica gel column chromatography steps using CH_2_Cl_2_/acetone (1:0 to 0:1, v/v), EtOAc/MeOH (10:1, v/v), and CH_2_Cl_2_/MeOH (6:1, v/v) as eluents.

### Spectroscopic data

Cornepalenin A (**1**): colorless needle crystal; ${[\alpha ]}_{D}^{25}$ = + 7.66 (*c* 0.29, MeOH); UV (MeOH) λ_max_ (log *ε*): 195 (3.24) nm; ECD (MeOH) *λ*_max_ (Δ*ɛ*) 197 (0.72); ^1^H (MeOH, 600 MHz) and ^13^C NMR (MeOH, 150 MHz) data, see Table 1; HRESIMS: *m*/*z* 335.1101 [M + Na] ^+^ (calc. for C_15_H_20_O_7_ Na^+^, 335.1111). Crystal data for cornepalenin A (**1**). C_15_H_20_O_7_ (M = 312.31 g/mol): monoclinic, space group P1 21 1, *a* = 6.4320(5) Å, *b* = 15.7546(11) Å, *c* = 7.0332(5) Å, *V* = 689.51(9) Å^3^, Z = 2, T = 100 K, *μ* (Cu K*α*) = 1.012 mm^−1^, *Dcalc* = 1.504 g/cm^3^, 13351 reflections measured (8.81^°^ ≤ *θ* ≤ 77.54^°^), 2806 unique (*R*_*int*_ = 0.0273, *R*_*sig*_ = 0.0253) which were used in all calculations. The final *R*_1_ was 0.0300 (I > 2*σ*(I)) and *wR*_2_ was 0.0768 (all data). The Flack parameter was 0.01(5). Crystallization solvent was CHCl_3_/MeOH. CCDC: 2421988 (https://www.ccdc.cam.ac.uk).

Cornepalenin B (**2**): colorless needle crystal**;** ${[\alpha ]}_{D}^{25}$= −5.00 (*c* 0.18, MeOH); UV (MeOH) *λ*_max_ (log *ε*): 198 (1.46) nm; ECD (MeOH) *λ*_max_ (Δ*ɛ*) 195 (3.94); ^1^H (CDCl_3_, 600 MHz) and ^13^C NMR (CDCl_3_, 150 MHz) data, see Table 1; HRESIMS: *m/z* 303.1209 [M + Na] ^+^ (calc. for C_15_H_20_O_5_Na^+^, 303.1203). Crystal data for cornepalenin B (**2**). C_15_H_20_O_5_ (M = 280.31 g/mol): monoclinic, space group P1 21 1, *a* = 8.8320 (4) Å, *b* = 13.0455 (6) Å, *c* = 12.4222(6) Å, *V* = 1385.88(11) Å^3^, Z = 4, T = 100 K, *μ* (Cu K*α*) = 0.082 mm^−1^, *Dcalc* = 1.343 g/cm^3^, 44062 reflections measured (3.67^°^ ≤ *θ* ≤ 77.90^°^), 5806 unique (*R*_*int*_ = 0.0320, *R*_*sig*_ = 0.0196) which were used in all calculations. The final *R*_1_ was 0.0270 (I > 2*σ*(I)) and *wR*_2_ was 0.0692 (all data). The Flack parameter was 0.10(2). Crystallization solvent was acetone. CCDC: 2421991 (https://www.ccdc.cam.ac.uk).

Cornepalenin C (**3**): colorless needle crystal; ${[\alpha ]}_{D}^{25}$ = + 125.0 (*c* 0.20, MeOH); UV (MeOH) *λ*_max_ (log *ε*): 195 (3.15) nm; ECD (MeOH) λ_max_ (Δ*ɛ*) 220 (5.87); ^1^H (MeOH, 600 MHz) and ^13^C NMR (MeOH, 150 MHz) data, see Table 1; HRESIMS: *m*/*z* 333.0936 [M + Na] ^+^ (calc. for C_15_H_18_O_7_Na^+^, 333.0945). Crystal data for cornepalenin C (**3**). C_15_H_18_O_7_ (M = 310.29 g/mol): monoclinic, space group P1 21 1, *a* = 9.1814(15) Å, *b* = 7.9983(13) Å, *c* = 9.5635(15) Å, *V* = 669.81(19) Å^3^, Z = 2, T = 100 K, *μ* (Cu K*α*) = 1.042 mm^−1^, *Dcalc* = 1.539 g/cm^3^, 17939 reflections measured (5.05^°^ ≤ *θ* ≤ 77.61^°^), 2809 unique (*R*_*int*_ = 0.0286, *R*_*sig*_ = 0.0235) which were used in all calculations. The final *R*_1_ was 0.0302 (I > 2*σ*(I)) and *wR*_2_ was 0.0759 (all data). The Flack parameter was 0.04(8). Crystallization solvent was CHCl_3_/MeOH. CCDC: 2421993 (https://www.ccdc.cam.ac.uk).

Cornepalenin D (**4**): colorless needle crystal; ${[\alpha ]}_{D}^{25}$ = + 22.37 (*c* 0.11, MeOH); UV (MeOH) *λ*_max_ (log ε): 195 (3.0) nm; ECD (MeOH) *λ*_max_ (Δ*ɛ*) 220 (3.96); ^1^H (CDCl_3_, 600 MHz) and ^13^C NMR (CDCl_3_, 150 MHz) data, see Table 1; HRESIMS: *m*/*z* 401.1207 [M + Na] ^+^ (calc. for C_19_H_22_O_8_Na^+^, 401.1204). Crystal data for cornepalenin D (**4**). C_19_H_22_O_8_ (M = 378.36 g/mol): monoclinic, space group P1 21 1, *a* = 10.4039(16) Å, *b* = 7.8149(12) Å, *c* = 11.2410(17) Å, *V* = 904.0(2) Å^3^, Z = 2, T = 100 K, *μ* (Cu K*α*) = 0.919 mm^−1^, *Dcalc* = 1.390 g/cm^3^, 23630 reflections measured (4.30^°^ ≤ *θ* ≤ 77.79^°^), 3780 unique (*R*_*int*_ = 0.0358, *R*_*sig*_ = 0.0287) which were used in all calculations. The final *R*_1_ was 0.0346 (I > 2*σ*(I)) and *wR*_2_ was 0.0982 (all data). The Flack parameter was −0.04(4). Crystallization solvent was CHCl_3_/MeOH. CCDC: 2421992 (https://www.ccdc.cam.ac.uk).

### ECD calculation

Calculated ECD spectra were generated based on Boltzmann weighting and UV offset [28]. Our previous study, involved conducting a conformation search using the MMFF94s force field through CONFLEX 8 following the geometry optimization of each candidate compound which possessed special configuration. The highest distribution of each conformer owning a relative energy within 10 kcal/mol was optimized using density functional theory (DFT) method at B3LYP/6-31G (d) level with the IEFPCM solvent model in MeOH by Gaussian 16 package of programs. Subsequently, the ECD calculations of optimized conformers were conducted using with DFT-B3LYP/6-311 + G (2 d, p) level under the same solvent model. The computational ECD spectra were generated using SpecDis 1.71 based on the Boltzmann weighting and UV shift.

### Primary CGNs culture

The 4–6-day-old C57BL/6 neonatal mice were obtained from Kunming Medical University. After the mice were euthanized, their cerebellar tissues were dissected. The tissue was gently dissociated using fire-polished pasteur pipettes and filtered through a cell strainer before being plated onto culture dishes pre-coated with poly-L-ornithine. Cells were cultured in DMEM supplemented with 5 mM Hepes buffer and 10% heat-inactivated FBS. After 5 days of culture, the cell density reached 90% [29].

### Cell culture and transfection

HEK-293 T cells were cultured in Dulbecco’s Modified Eagle’s Medium (DMEM) containing 10% FBS and seeded in 6-well plates. When the cell density reached 70–90%, the plasmids GABBR1 and GABBR2 were co-transfected into the cells using the Lip3000 transfection reagent [29].

### Intracellular calcium release measurements

Intracellular calcium release measurements were performed as previously described. Briefly, CGNs and HEK-293 T cells were washed twice with PBS buffer and then loaded with the calcium-sensitive dye Fluo-4 AM (2 μM) [29]. After incubation at 37 °C for 1 h, the dye was aspirated, and the cells were washed twice with PBS. Pre-prepared drug solutions were added to each well and incubated for 1 h. Subsequently, pre-prepared agonist solutions were added and incubated for another hour. Fluorescence intensity data were then measured using an inverted fluorescence microscope.

### Drug treatments and Western blotting

Drug treatments and Western blotting experiments [30] were performed to detect the activation of AKT and ERK1/2. For the treatment with compound **6**, cell cultures were pretreated with compound **6** for 60 min, followed by stimulation with freshly prepared baclofen or GABA. At the end of the treatment, the cells were quickly washed with ice-cold Ca^2^⁺-free PBS (pH 7.4), 200 μL of lysis buffer was added, and the cells were immediately placed on ice. Equal amounts of protein (20 μg) were resolved by SDS/10% PAGE. Proteins were transferred to nitrocellulose membranes (Millipore) and blocked in protein-free rapid blocking buffer for 15 min at room temperature. The blots were then incubated with primary antibodies at the relevant dilution (Cell Signaling Technology) overnight at 4 °C, and with HRP-linked secondary antibodies (1:1000; Bi Yun Tian) for 2 h. Immunoblots were detected by using enhanced chemiluminescence reagents (Pierce) and visualized using X-ray film. The density of immunoreactive bands was measured using National Institutes of Health (NIH) image software, and all bands were normalized to the percentages of the control values.

### Statistical analysis

Graphs and statistical analysis were performed using GraphPad Prism software (Version 5.0; GraphPad Software Inc.). The IC_50_ values were determined using non-linear regression and a three-parameter logistic equation. Statistical significance between different groups was calculated using Student’s t-test.

## Supplementary Information

Supplementary material 1.

## Data Availability

All data generated or analyzed during this study are available in this published article and its Additional files.
