# Supplementary material for: Cornepalenins A–D, picrotoxane-type sesquiterpenes from the flowers of Coriaria nepalensis associated with modulation of GABAB receptor–related signaling
Source: Nat Prod Bioprospect. 2026 Aug 24;16(1):83. doi: 10.1007/s13659-026-00623-1 (PMC13504047; doi:10.1007/s13659-026-00623-1)
Supplement: Supplementary file 1 [file 13659_2026_623_MOESM1_ESM.docx]

**Supporting Information**

**Cornepalenins A-D, picrotoxane-type sesquiterpenes from the flowers of *Coriaria nepalensis* with inhibitory effects on GABA_B_ receptor**

Min Tan^1,2^, Rong Xie^1^, Dan Liu^1^, Yao Bai^1^, Zhi-Jun Zhang^1,^*, Rong-Tao Li^1,2^*

^1^Faculty of Life Science and Technology, Kunming University of Science and Technology, Kunming 650500, Yunnan, China

^2^Southwest United Graduate School, Kunming 650092, Yunnan, China

*Corresponding author.

E-mail address: zhangzj@kust.edu.cn (Z.J. Zhang); rongtaolikm@163.com (R.T. Li).

**Table of contents**

The ^1^H and ^13^C NMR data of compounds **5**–**11**	4

[Figure S1. Key ROESY correlation of compound **1**–**4**.	6](#_Toc160656082)

[Figure S2. Experimental and calculated ECD spectra compounds **1**–**4**	6](#_Toc160656084)

[Figure S3. GABA_B_ receptor overexpression system in HEK-293T cells	6](#_Toc160656099)

[Figure S4. HRESIMS of **1**	7](#_Toc160656103)

[Figure S5. ^1^H NMR spectrum of **1** (600 MHz, CD_3_OD)	8](#_Toc160656103)

[Figure S6. ^13^C NMR and DEPT spectrum of **1** (150 MHz, CD_3_OD)	8](#_Toc160656104)

[Figure S7. HSQC spectrum of **1**	9](#_Toc160656105)

[Figure S8. HMBC spectrum of **1**	9](#_Toc160656106)

[Figure S9. ^1^H-^1^H COSY spectrum of **1**	10](#_Toc160656107)

[Figure S10. ROESY spectrum of **1**	10](#_Toc160656108)

[Figure S11. HR-ESI-MS spectrum of **2**	11](#_Toc160656109)

[Figure S12. ^1^H NMR spectrum of **2** (600 MHz, CDCl_3_)	12](#_Toc160656111)

[Figure S13. ^13^C and DEPT NMR spectrum of **2** (150 MHz, CDCl_3_)	12](#_Toc160656112)

[Figure S14. HSQC spectrum of **2**	13](#_Toc160656113)

[Figure S15. HMBC spectrum of **2**	13](#_Toc160656114)

[Figure S16. ^1^H-^1^H COSY spectrum of **2**	14](#_Toc160656115)

[Figure S17. ROESY spectrum of **2**	14](#_Toc160656117)

[Figure S18. HR-ESI-MS spectrum of **3**	15](#_Toc160656118)

[Figure S19. ^1^H NMR spectrum of **3** (600 MHz, CD_3_OD)	16](#_Toc160656120)

[Figure S20. ^13^C NMR and DEPT spectrum of **3** (150 MHz, CD_3_OD)	16](#_Toc160656121)

[Figure S21. HSQC spectrum of](#_Toc160656123) **[3](#_Toc160656123)**	[17](#_Toc160656123)

[Figure S22. HMBC spectrum of **3**	17](#_Toc160656124)

[Figure S23. ^1^H-^1^H COSY spectrum of **3**	18](#_Toc160656125)

[Figure S24. ROESY spectrum of **3**	18](#_Toc160656126)

[Figure S25. HR-ESI-MS spectrum of](#_Toc160656128) **[4](#_Toc160656128)**	[19](#_Toc160656128)

[Figure S26. ^1^H NMR spectrum of **4** (600 MHz, CDCl_3_)..	20](#_Toc160656130)

[Figure S27. ^13^C NMR and DEPT spectrum of **4** (150 MHz, CDCl_3_).	20](#_Toc160656131)

[Figure S28. HSQC spectrum of **4**	21](#_Toc160656123)

[Figure S29. HMBC spectrum of **4**	21](#_Toc160656124)

[Figure S30. ^1^H-^1^H COSY spectrum of **4**	22](#_Toc160656125)

[Figure S31. ROESY spectrum of **4**	22](#_Toc160656126)

[Figure S32. Effect of compound **1** on Ca²⁺ release in CGNs at different concentration ......... 23](#_Toc160656130)

[Figure S33. Effect of compound **5** on Ca²⁺ release in CGNs at different concentration .......... 23](#_Toc160656130)

[Figure S34. Effect of compound **6** on Ca²⁺ release in CGNs at different concentration..	23](#_Toc160656130)

[Figure S35. Effect of compounds **1**-**11** on Ca²⁺ release in HEK-293T cells under GABA (5 *μ*M) stimulation..	24](#_Toc160656130)

[Figure S36. Effect of compounds **1**-**11** on Ca²⁺ release in HEK-293T cells under Baclofen (1 *μ*M) stimulation..	24](#_Toc160656130)

[Figure S37. Effect of CGP54626 at different concentrations on Ca²⁺ release in HEK-293T cells under GABA (5 *μ*M) stimulation..	24](#_Toc160656130)

[Figure S38. Effect of CGP54626 at different concentrations on Ca²⁺ release in HEK-293T cells under Baclofen (1 *μ*M) stimulation..	25](#_Toc160656130)

[Figure S39. Effect of **1** at different concentrations on Ca²⁺ release in HEK-293T cells under GABA (5 *μ*M) stimulation..	25](#_Toc160656130)

[Figure S40. Effect of **1** at different concentrations on Ca²⁺ release in HEK-293T cells under Baclofen (1 *μ*M) stimulation..	25](#_Toc160656130)

[Figure S41. Effect of **5** at different concentrations on Ca²⁺ release in HEK-293T cells under GABA (5 *μ*M) stimulation..	26](#_Toc160656130)

[Figure S42. Effect of **5** at different concentrations on Ca²⁺ release in HEK-293T cells under Baclofen (1 *μ*M) stimulation..	26](#_Toc160656130)

[Figure S43. Effect of **6** at different concentrations on Ca²⁺ release in HEK-293T cells under GABA (5 *μ*M) stimulation..	26](#_Toc160656130)

[Figure S44. Effect of **6** at different concentrations on Ca²⁺ release in HEK-293T cells under Baclofen (1 *μ*M) stimulation..	27](#_Toc160656130)

[Figure S45. Effect of **9** at different concentrations on Ca²⁺ release in HEK-293T cells under GABA (5 *μ*M)	stimulation..	27](#_Toc160656130)

[Figure S46. Effect of **9** at different concentrations on Ca²⁺ release in HEK-293T cells under Baclofen (1 *μ*M) stimulation..	27](#_Toc160656130)

[Figure S47. 2D and 3D representations of compound **2**, **4, 7** and **10** showing their interactions with the GABA_A_ receptor..	28](#_Toc160656130)

[Figure S48. 2D and 3D representations of compound **2**, **4** showing their interactions with the GABA_B_ receptor..	29](#_Toc160656130)

[Figure S49. 2D and 3D representations of compound **7**, **10** showing their interactions with the GABA_B_ receptor..	30](#_Toc160656130)

[Figure S50. Effect of compound **6** on AKT1 phosphorylation induced by GABA or baclofen in transfected HEK 293T cells ...	31](#_Toc160656130)

# The ^1^H and ^13^CNMR data of compounds 5—11

**Coriamyrtin** (**5**): C_15_H_18_O_5_, colorless oil, ^1^H NMR (600 MHz, CDCl_3_): *δ*_H_ 4.98 (1H, s, H-9a), 4.88 (1H, t, *J* = 4.2 Hz, H-9b), 4.66 (1H, s, H-3), 3.76 (1H, d, *J* = 3.0 Hz, H-11), 3.27 (1H, d, *J* = 3.0 Hz, H-12), 3.15 (1H, d, *J* = 4.3 Hz, H-5), 3.08 (1H, d, *J* = 3.7 Hz, H-14a), 3.06 (1H, s, H-4), 2.90 (1H, d, *J* = 3.7 Hz, H-14b), 1.94 (3H, s, H_3_-10), 1.79 (1H, d, *J* = 15.9 Hz, H-2a), 1.66 (1H, d, *J* = 16.0 Hz, H-2b), 1.15 (3H, s, H_3_-7). ^13^C NMR (150 MHz, CDCl_3_): *δ*_C_ 174.9 (C-15), 141.5 (C-8), 111.8 (C-9), 78.6 (C-3), 76.5 (C-6), 65.9 (C-13), 61.0 (C-11), 59.0 (C-12), 52.1 (C-14), 48.6 (C-5), 48.1 (C-4), 39.2 (C-1), 30.6 (C-2), 23.1 (C-7), 22.5 (C-10).

**Tutin** (**6**): C_15_H_18_O_6_, colorless needle crystal, ^1^H NMR (600 MHz, Acetone): *δ*_H_ 4.85 (1H, s, H-3), 4.84 (1H, s, H-9a), 4.78 (1H, s, 2-OH), 4.67 (1H, s, H-9b), 4.23 (1H, d, *J* = 5.3 Hz, H-2), 3.97 (1H, d, *J* = 6.2 Hz, H-14a), 3.67 (1H, d, *J* = 3.1 Hz, H-11), 3.25 (1H, s, H-4), 3.12 (1H, d, *J* = 4.1 Hz, H-5), 3.04 (1H, d, *J* = 3.2 Hz, H-12), 2.62 (1H, d, *J* = 6.2 Hz, H-14b), 1.94 (3H, s, H_3_-10), 1.33 (3H, s, H_3_-7). ^13^C NMR (150 MHz, Acetone): *δ*_C_ 174.7 (C-15), 142.4 (C-8), 109.5 (C-9), 83.4 (C-3), 77.4 (C-6), 72.0 (C-2), 65.0 (C-13), 60.2 (C-11), 59.4 (C-12), 51.1 (C-14), 49.6 (C-5), 49.2 (C-4), 44.9 (C-1), 22.3 (C-7), 20.0 (C-10).

**Corianlactone** (**7**): C_15_H_16_O_6_, colorless oil, ^1^H NMR (600 MHz, Acetone): *δ*_H_ 4.38 (1H, dd, *J* = 10.6, 8.1 Hz, H-9a), 4.30 (1H, dd, *J* = 10.6, 2.0 Hz, H-9b), 3.97 (1H, d, *J* = 3.1 Hz, H-11), 3.80 (1H, d, *J* = 11.6 Hz, H-5), 3.77 (1H, d, *J* = 5.2 Hz, H-14a), 3.38 (1H, d, *J* = 3.1 Hz, H-12), 3.22–3.13 (1H, m, H-4), 2.89 (1H, s, H-14b), 2.86 (1H, s, H-3a), 2.44 (1H, d, *J* = 17.2 Hz, H-3b), 1.49 (3H, s, H_3_-7), 1.49 (3H, s, H_3_-10). ^13^C NMR (150 MHz, Acetone): *δ*_C_ 202.6 (C-2), 173.1 (C-15), 89.0 (C-6), 87.3 (C-8), 66.3 (C-9), 61.7 (C-13), 57.6 (C-11), 56.2 (C-12), 54.3 (C-5), 50.9 (C-1), 50.3 (C-14), 49.9 (C-4), 43.6 (C-3), 23.7 (C-10), 21.8 (C-7).

**Hyenanchin** (**8**): C_15_H_18_O_7_, colorless needle crystal , ^1^H NMR (600 MHz, CD_3_OD): *δ*_H_ 4.94 (1H, s, H-9a), 4.82 (1H, s, H-9b), 4.59 (1H, s, H-3), 4.09 (1H, s, H-2), 3.94 (1H, d, *J* = 5.7 Hz, H-14a), 3.61 (1H, d, *J* = 3.0 Hz, H-11), 2.99 (1H, d, *J* = 2.9 Hz, H-12), 2.85 (1H, s, H-5), 2.65 (1H, d, *J* = 5.7 Hz, H-14b), 1.88 (3H, s, H_3_-7), 1.22 (3H, s, H_3_-10). ^13^C NMR (150 MHz, CD_3_OD): *δ*_C_ 177.2 (C-15), 145.5 (C-8), 113.2 (C-9), 90.1 (C-3), 81.1 (C-4), 78.5 (C-6), 76.0 (C-2), 67.0 (C-13), 61.6 (C-11), 60.6 (C-12), 58.4 (C-5), 52.9 (C-14), 45.3 (C-1), 20.9 (C-10), 20.5 (C-7).

**Corianin** (**9**): C_15_H_18_O_6_, colorless needle crystal, ^1^H NMR (600 MHz, Acetone): *δ*_H_ 5.02 (1H , t, *J* = 4.2 Hz, H-3), 4.94 (2H, s, H-9), 4.30 (1H, s, 2-OH), 4.23 (1H, d, *J* = 4.2 Hz, H-2), 4.19 (d, *J* = 1.8 Hz, H-11), 3.89 (dd, *J* = 10.3, 1.8 Hz, H-14a), 3.80 (1H, d, *J* = 10.2 Hz, H-14b), 3.77 (1H, d, *J* = 2.6 Hz, H-12), 3.50 (1H, d, *J* = 2.4 Hz, H-4), 3.14 (1H, d, *J* = 4.4 Hz, H-5), 1.96 (3H, s, H_3_-10), 1.08 (3H, s, H_3_-7). ^13^C NMR (150 MHz, Acetone): *δ*_C_ 175.4 (C-15), 141.5 (C-8), 112.6 (C-9), 90.7 (C-13), 85.7 (C-3), 82.1 (C-2), 77.7 (C-14), 75.9 (C-6), 64.3 (C-11), 61.1 (C-12), 55.5 (C-1), 50.0 (C-5), 49.1 (C-4), 23.1 (C-7), 21.9 (C-10).

**Acetylcorianin** (**10**): C_17_H_20_O_7_, colorless oil, ^1^H NMR (600 MHz, CD_3_OD): *δ*_H_ 5.01 (1H, s, H-3), 4.87 (1H, s, H-9a),4.80 (1H, s, H-9b), 4.23 (1H, d, *J* = 3.7 Hz, H-2), 4.17 (1H, d, *J* = 11.8 Hz, H-14a), 3.96 (1H, d, *J* = 2.3 Hz, H-11), 3.94 (1H, d, *J* = 11.8 Hz, H-14b), 3.80 (1H, d, *J* = 2.3 Hz, H-12), 3.31 (1H, s, H-5), 3.11 (1H, d, *J* = 4.2 Hz, H-4), 2.03 (3H, s, OAc), 1.89 (3H, s, H_3_-10), 1.10 (3H, s, H_3_-7). ^13^C NMR (150 MHz, CD_3_OD): *δ*_C_ 176.7 (C-15), 172.2 (C-13′), 141.7 (C-8), 113.1 (C-9), 97.2 (C-13), 85.6 (C-3), 82.7 (C-2), 75.6 (C-14), 75.2 (C-6), 65.7 (C-11), 58.7 (C-12), 57.5 (C-1), 50.6 (C-15), 48.6 (C-4), 23.1 (C-7), 22.4 (C-10), 21.2 (C-13′′).

**Acetyltutin** (**11**): C_17_H_20_O_7_, colorless oil, ^1^H NMR (600 MHz, CD_3_OD): *δ*_H_ 5.40 (1H, s, H-2), 5.06 (1H, s, H-9a), 4.92–4.89 (2H, m, H-9b, H-3,), 3.76 (d, *J* = 5.1 Hz, H-14a), 3.72 (d, *J* = 3.0 Hz, H-11), 3.45 (1H, s, H-4), 3.16 (1H, d, *J* = 4.2 Hz, H-5), 3.13 (1H, d, *J* = 3.0 Hz, H-12), 2.82 (1H, d, *J* = 5.1 Hz, H-14b), 2.08 (3H, s, OAc), 1.94 (3H, s, H_3_-10), 1.21 (3H, s, H_3_-7). ^13^C NMR (150 MHz, CD_3_OD): *δ*_C_ 176.8 (C-15), 172.1 (C-2′), 142.9 (C-8), 111.6 (C-9), 81.8 (C-3), 78.6 (C-6), 75.8 (C-2), 66.4 (C-13), 61.3 (C-11), 60.3 (C-12), 53.3 (C-14), 50.9 (C-4), 50.9 (C-5), 46.0 (C-1), 23.2 (C-10), 20.7 (C-2′′), 20.5 (C-7).

**Figure S1**. Key ROESY correlation of compound **1**–**4**.

**Figure S2**. Experimental and calculated ECD spectra compounds **1**–**4**.

**Figure S3**. GABA_B_ receptor overexpression system in HEK-293T cells.

**Figure S4**. HRESIMS of compound **1**.

**Figure S5**. ^1^H-NMR spectrum (CD_3_OD, 600 MHz) of compound **1**.

**Figure S6**. ^13^C and DEPT NMR spectra (CD_3_OD, 150 MHz) of compound **1**.

**Figure S7**. HSQC spectrum of compound **1**.

**Figure S8**. HMBC spectrum of compound **1**.

**Figure S9**. ^1^H-^1^H COSY spectrum of compound **1**.

**Figure S10**. ROESY spectrum of compound **1**.

**Figure S11**. HRESIMS of compound **2**.

**Figure S12**. ^1^H-NMR spectrum (CDCl_3_, 600 MHz) of compound **2**.

**Figure S13**. ^13^C and DEPT NMR spectra (CDCl_3_, 150 MHz) of compound **2**.

**Figure S14**. HSQC spectrum of compound **2**.

**Figure S15**. HMBC spectrum of compound **2**.

**Figure S16**. ^1^H-^1^H COSY spectrum of compound **2**.

**Figure S17**. ROESY spectrum of compound **2**.

**Figure S18**. HRESIMS of compound **3**.

**Figure S19**. ^1^H-NMR spectrum (CDCl_3_, 600 MHz) of compound **3**.

**Figure S20**. ^13^C and DEPT NMR spectra (CDCl_3_, 150 MHz) of compound **3**.

**Figure S21**. HSQC spectrum of compound **3.**

**Figure S22**. HMBC spectrum of compound **3**.

**Figure S23**. ^1^H-^1^H COSY spectrum of compound **3**.

**Figure S24**. ROESY spectrum of compound **3**.

**Figure S25**. HRESIMS of compound **4**.

**Figure S26**. ^1^H-NMR spectrum (CD_3_OD, 600 MHz) of compound **4**.

**Figure S27**. ^13^C and DEPT NMR spectra (CD_3_OD, 150 MHz) of compound **4**.

**Figure S28**. HSQC spectrum of compound **4**.

**Figure S29**. HMBC spectrum of compound **4**.

**Figure S30**. ^1^H-^1^H COSY spectrum of compound **4**.

**Figure S31**. ROESY spectrum of compound **4**.

**Figure** **S32**. Effect of compound **1** on Ca²⁺ release in CGNs at different concentrations.

**Figure** **S33**. Effect of compound **5** on Ca²⁺ release in CGNs at different concentrations.

**Figure** **S34**. Effect of compound **6** on Ca²⁺ release in CGNs at different concentrations.

**Figure** **S35**. Effect of compounds **1**-**11** on Ca²⁺ release in HEK-293T cells under

GABA (5 *μ*M) stimulation.

**Figure** **S36**. Effect of compounds **1**-**11** on Ca²⁺ release in HEK-293T cells under

Baclofen (1 *μ*M) stimulation.

**Figure** **S37**. Effect of CGP54626 at different concentrations on Ca²⁺ release in

HEK-293T cells under GABA stimulation.

**Figure** **S38**. Effect of CGP54626 at different concentrations on Ca²⁺ release in

HEK-293T cells under baclofen stimulation.

**Figure** **S39**. Effect of **1** at different concentrations on Ca²⁺ release in HEK-293T cells under GABA stimulation.

**Figure** **S40**. Effect of **1** at different concentrations on Ca²⁺ release in HEK-293T cells under baclofen stimulation.

**Figure** **S41**. Effect of **5** at different concentrations on Ca²⁺ release in HEK-293T cells under GABA stimulation.

**Figure** **S42**. Effect of **5** at different concentrations on Ca²⁺ release in HEK-293T cells under baclofen stimulation.

**Figure** **S43**. Effect of **6** at different concentrations on Ca²⁺ release in HEK-293T cells under GABA stimulation.

**Figure** **S44**. Effect of **6** at different concentrations on Ca²⁺ release in HEK-293T cells under baclofen stimulation.

**Figure** **S45**. Effect of **9** at different concentrations on Ca²⁺ release in HEK-293T cells under GABA stimulation.

**Figure** **S46**. Effect of **9** at different concentrations on Ca²⁺ release in HEK-293T cells under baclofen stimulation.

**Figure** **S47**. 2D and 3D representations of compound **2**, **3**, **7** and **10** showing their interactions with the GABA_A_ receptor.

**Figure** **S48**. 2D and 3D representations of compound **2**, **3** showing their interactions with the GABA_B_ receptor.

**Figure** **S49**. 2D and 3D representations of compound **7**, **10** showing their interactions with the GABA_B_ receptor.

**Figure** **S50**. Effect of compound **6** on AKT1 phosphorylation induced by GABA or baclofen in transfected HEK 293T cells.
